# *Rheum rhaponticum* Root Extract Improves Vasomotor Menopausal Symptoms and Estrogen-Regulated Targets in Ovariectomized Rat Model

**DOI:** 10.3390/ijms22031032

**Published:** 2021-01-21

**Authors:** Mickey Wilson, Veera Konda, Kathryn Heidt, Thirumurugan Rathinasabapathy, Anuradha Desai, Slavko Komarnytsky

**Affiliations:** 1 Plants for Human Health Institute, North Carolina Research Campus, North Carolina State University, 600 Laureate Way, Kannapolis, NC 28081, USA; mlwilso8@ncsu.edu (M.W.); ksheidt@catawba.edu (K.H.); trathin@ncsu.edu (T.R.); 2 Department of Food, Bioprocessing & Nutrition Sciences, North Carolina State University, 400 Dan Allen Drive, Raleigh, NC 27695, USA; 3 Metagenics Inc., 9770 44th Ave NW, Gig Harbor, WA 98332, USA; veerakonda@gmail.com (V.K.); AnuDesai@metagenics.com (A.D.); 4 Department of Biology, Catawba College, 2300 W Innes Street, Salisbury, NC 28144, USA

**Keywords:** postmenopausal, estrogens, hot flashes, HRT, ERr 731

## Abstract

Ovarian insufficiency and ovariectomy are characterized by deregulated heat loss mechanisms. Unlike hormone therapy, ERr 731 (a standardized botanical extract of Siberian rhubarb *Rheum rhaponticum* L. high in rhaponticin) acts like a selective estrogen receptor modulator for ERβ receptors and may offer a higher degree of safety while maintaining the desired efficacy profile. In this study, we examined the relationship between oral administration of ERr 731 and the underlying components of skin vasomotion responses in an ovariectomized (OVX) rat model. ERr 731 dose-dependently reduced tail skin temperature (T_skin_) values by an average of 1 °C. The rapid onset of this effect was observed in 1 and 3 mg/kg/day ERr 731 groups as early as day 2 of administration, and remained in place for the duration of the treatment (2 weeks). Substituting ERr 731 after E_2_ withdrawal helped maintain body temperature similarly to E_2_ alone, suggesting the usefulness of ERr 731 for replacing existing hormonal therapy in humans. ERr 731 also acted as a highly selective agonist for ERβ in the hypothalamus of OVX rats, as well as in ERα/β cell-based reporter assays. These data validate the OVX/T_skin_ rat model as a suitable screening platform to evaluate botanical and pharmaceutical treatments of menopause, while providing further evidence for the efficacy of ERr 731 towards alleviating vasomotor menopausal symptoms and improving wellbeing during the menopausal transition.

## 1. Introduction

An estimated 6000 American women transition to menopause every day, with 75% of women aged 50 to 55 years old assumed to be postmenopausal [1]. Among the eleven key health issues selected in terms of burden exerted in women’s mortality, morbidity, disability and quality of life, vasomotor symptoms play a prominent role [2]. To date, the most effective and reliable therapy for menopausal vasomotor symptoms (e.g., hot flushes, night sweats) is hormone replacement therapy (HRT). However, long-term HRT carries the increased risk of hormone-related cancers and cardiovascular disease [3]. Mood modulators and selective serotonin re-uptake inhibitors are commonly recommended for short-term use in highly symptomatic women who are not HRT candidates, but these treatments lack supported data on safety and efficacy [4]. Natural approaches for hot flush relief aim to reduce the daily vasomotor symptoms (frequency, intensity, and number) and improve quality of life through supplementation with phytoestrogens (soy, red clover, flax, hops, hesperidin, and kudzu) and non-phytoestrogens, i.e., black cohosh, essential fatty acids, vitamin E, and succinates [5]. Unlike HRT, these approaches may specifically target specific estrogen receptors (ER) and therefore offer more selective clinical benefits [6].

**Figure 1:**
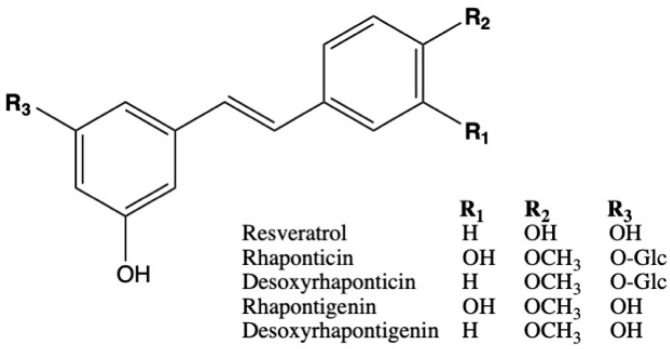
Schematic representation of bioactive constituents from the ERr 731 rhubarb root extract in comparison to their respective glycosides and trans-resveratrol.

Rhapontic rhubarb roots (*Rheum rhaponticum* L., family Polygonaceae) contain a significant amount of hydroxystilbene compounds without any anthraquinones [7]. The standardized ERr 731 extract contains rhaponticin and desoxyrhaponticin glycosides as the main bioactive constituents (Figure 1), in addition to lesser amounts of their respective rhapontigenin and desoxyrhapontigenin aglycones (~5%) [8]. The ERr 731 extract, as well as its individual compounds, have been demonstrated to act as potent, selective ERβ agonists in human endometrial cells, without significant ERα effects [9]. The safety of ERr 731 was evaluated in 90-day rat [10] and 13-week dog [11] studies. Subsequent clinical investigations suggested that ERr 731 was well tolerated in the long-term observational studies [7,12]. ERr 731 was effective in decreasing the number and severity of hot flushes in a 12-week multicenter trial that included 109 women with climacteric complaints [7], a menopause rating scale total score given after 6 months of treatment in 252 women [13], as well as 112 perimenopausal women with menopausal symptoms followed for 12 weeks [14]. Postmarketing surveillance data from Germany (140 million daily doses), the United States and Canada (13 million) suggest that the extract is generally safe for consumption [15]. However, it is not clear how effective ERr 731 supplementation is following the substitution after termination of HRT (E_2_ withdrawal).

In rats, vasodilation of the tail is a primary heat-dissipating mechanism [16] that can be monitored by measuring tail skin temperature T_skin_ [17]. T_skin_ is increased by ovariectomy (OVX) and reduced by administration of 17β-estradiol (E_2_), thus making the OVX rat model combined with an inexpensive, non-invasive T_skin_ monitoring device attached in a protective covering on the surface of the tail a versatile screening platform to evaluate botanical and pharmaceutical treatments of hot flushes and general wellbeing during menopause [18]. Because hot flushes correlate to a spiking of the peripheral LH and GnRH released from the hypothalamus, they most likely represent a disorder of hypothalamic thermoregulation [19].

The present study was designed to examine the acute and replacement therapy effects of ERr 731 supplementation on several underlying components of skin vasomotion responses in the OVX rat model, and to substantiate these changes with the hypothalamic expression patterns of genes related to the ERα/β signaling and bioactive peptides.

## 2. Results

### 2.1. ERr 731 and E_2_ Treatments Lower T_skin_ in OVX Rat Model

**Figure 2:**
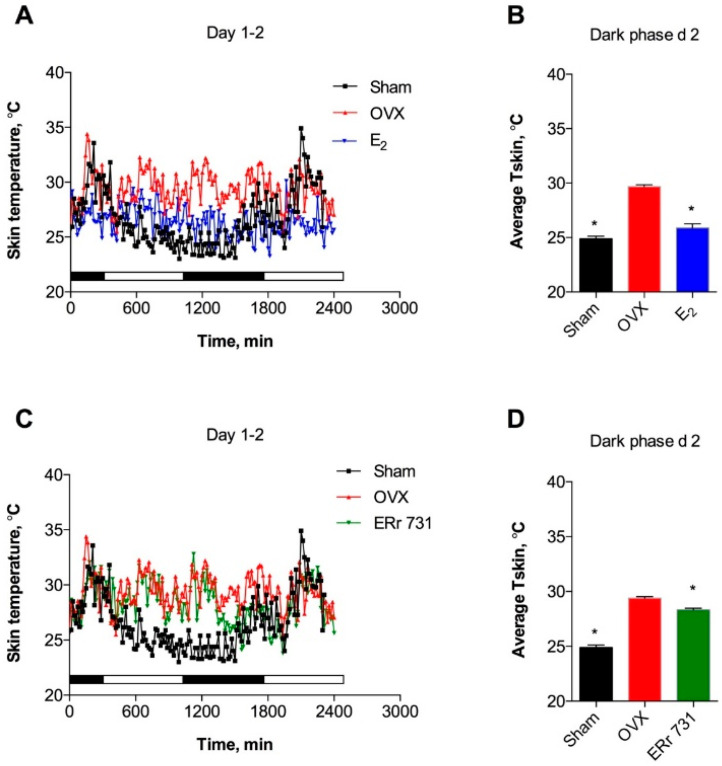
Effects of ERr 731 and 17β-estradiol (E_2_) on tail skin temperature (T_skin_) in ovariectomized (OVX) rat model after 2 days of treatment. (**A**) Recordings of OVX controls (red) show circadian rhythm and increased T_skin_ as compared to intact (Sham, black) animals, while E_2_ treatment (blue) is associated with decreased T_skin_ on days 1–2; (**B**) Average T_skin_ is significantly increased in OVX rats and is reduced following E_2_ treatment in the dark phase of day 2; (**C**) Recordings of decreased T_skin_ following ERr 731 treatment (green) on days 1–2; (**D**) Average T_skin_ is significantly reduced in ERr 731-treated rats in the dark phase of day 2. Data are the mean ± SEM (*n* = 6), * *p* < 0.05 vs. OVX control.

**Figure 3:**
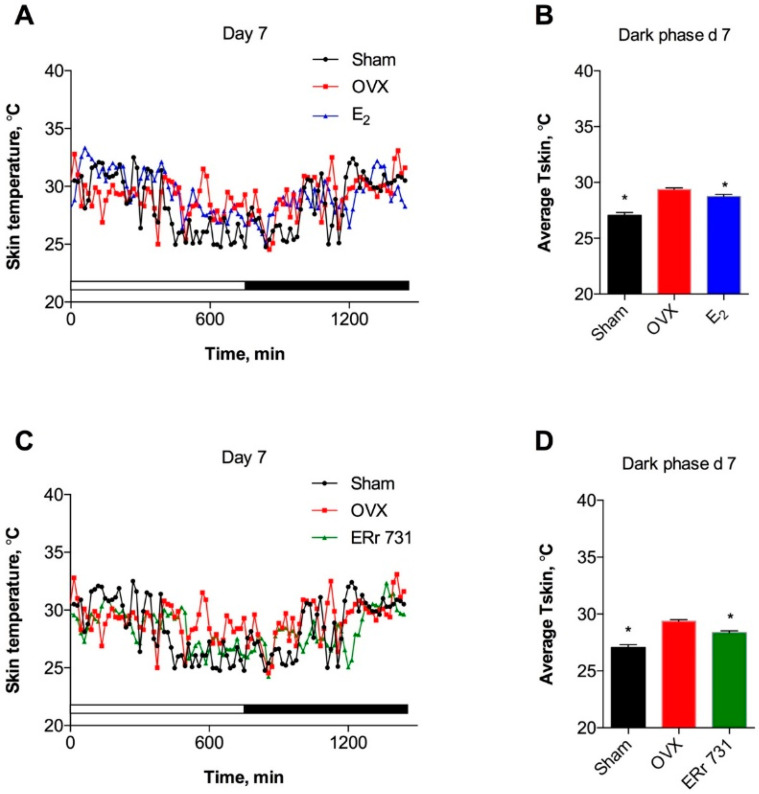
Effects of ERr 731 and E_2_ on T_skin_ persisted for one week of treatment. (**A**) Recordings of OVX controls (red) show circadian rhythm and increased T_skin_ as compared to intact (Sham, black) animals, while E_2_ treatment (blue) is associated with decreased T_skin_ on day 7; (**B**) Average T_skin_ is significantly increased in OVX rats and reduced following E_2_ treatment in the dark phase of day 7; (**C**) Recordings of decreased T_skin_ following ERr 731 treatment (green) on day 7; (**D**) Average T_skin_ is significantly reduced in ERr 731-treated rats in the dark phase of day 7. Data are the mean ± SEM (*n* = 6), * *p* < 0.05 vs. OVX control.

All treatment groups displayed circadian rhythms of T_skin_ with multiple spontaneous fluctuations. OVX animals showed a clear increase in T_skin_ over the respective Sham controls during both the dark (active) and light (inactive) phases of the day (Figure 2A). E_2_ treatment (0.1 mg/kg/day) of OVX rats resulted in decreased T_skin_ that was evident on day 1 and reached significance by the dark phase of day 2 (Figure 2B). ERr 731 treatment at 1 mg/kg/day produced a mild suppression of T_skin_ during the dark phase that was only significantly different from OVX rats on a few occasions, starting on day 2 (Figure 2C,D). The T_skin_-lowering effects of both treatments persisted for the duration of the experiment, as was evident on day 7 of the treatment (Figure 3A,D).

### 2.2. Dose-Dependent Effects of ERr 731 Treatment

**Figure 4:**
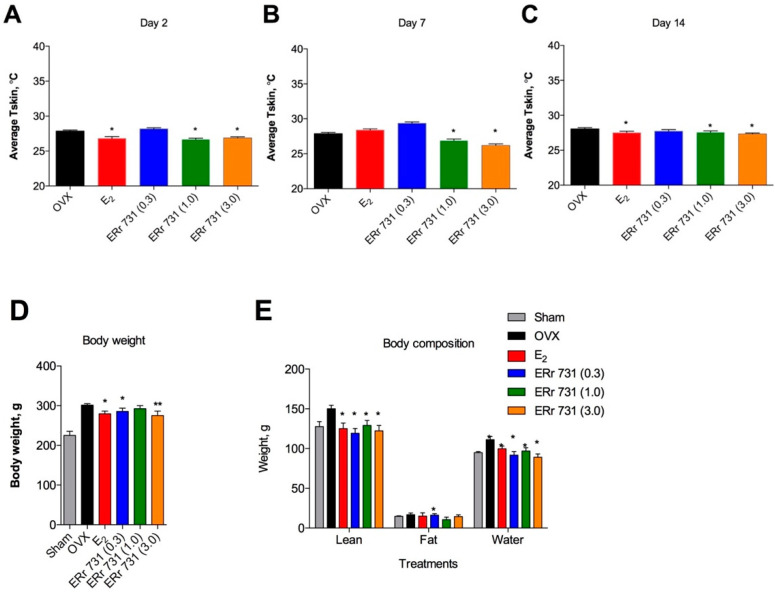
Dose-dependent effects of ERr 731. Average T_skin_ was significantly increased in OVX rats and was reduced following E_2_ (0.1 mg/kg/day) or ERr 731 (0.3 mg/kg/day, 1 mg/kg/day, and 3 mg/kg/day) oral supplementation for two weeks in the dark phase of (**A**) day 2, (**B**) day 7, and (**C**) day 14 of the study. (**D**) Compared with Sham animals, the average body weight was significantly increased in OVX rats and decreased in E_2_- and ERr 731-treated animals. (**E**) Body composition measurements indicated an equal reduction in lean fat mass and total body water in all treatments with the exception of animals receiving 1 mg/kg/day ERr 731, which showed a significant decrease in fat mass associated with the treatment. Data are the mean ± SEM (*n* = 6), * *p* < 0.05, ** *p* < 0.01 vs. OVX control.

The dose response effects of ERr 731 on T_skin_ were evident in the dark phase of days 2, 7 and 14 of the dose ranging study. E_2_ treatment (0.1 mg/kg/day) was significantly effective in reducing T_skin_ on days 2 and 14, but did not reach significance on day 7. ERr 731 administered at doses of 1 and 3 mg/kg/day lowered T_skin_ similar to E_2_. A lower dose of the ERr 731 extract (0.3 mg/kg/day) did not have a significant effect on T_skin_ over the 14-day treatment period (Figure 4A–C).

OVX surgery resulted in a significant body weight gain as compared to Shams, and this effect was partially reversed by E_2_ treatment and the highest dose of the ERr 731 extract. A similar, but non-significant, trend was observed with lower doses of the ERr 731 extract as well (Figure 4D). Body composition measurements showed that the observed decrease in total body weight was due to an equal reduction of lean fat mass and total body water. A significant decrease in fat mass was observed in animals receiving 1 mg/kg/day ERr 731, but this effect was not dose-dependent (Figure 4E).

### 2.3. Replacement Effects of ERr 731 in OVX Rats Receiving E_2_

**Figure 5:**
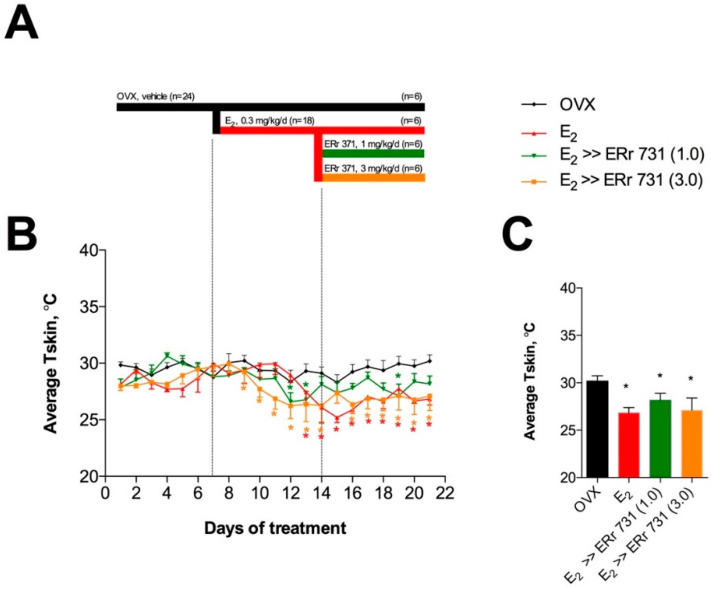
Time course of E_2_ supplementation and ERr 731 replacement therapy in OVX rats. (**A**) Schematic diagram of the replacement strategy. (**B**) In the dark phase, the E_2_ treatment of OVX rats significantly reduced T_skin_ from experimental day 10 (day 2 of E_2_ treatment) through the end of the study. In rats that received ERr 731 therapy, the effect remained significant at high doses (3 mg/kg/day) and reached significance on several days at middle dose (1 mg/kg/day) through the end of the study. (**C**) Overall average T_skin_ was significantly increased in OVX rats and reduced following either E_2_ (0.3 mg/kg/day) or ERr 731 replacement therapy (1–3 mg/kg/day) for the duration of the treatment. Data are the mean ± SEM (*n* = 6), * *p* < 0.05 vs. OVX control.

In modeling the ERr 731 replacement strategy, 24 animals in study 3 received daily gavage with vehicle during the initial phase of the study (black bar, days 1–7) for one week, to establish baseline T_skin_ averages. Next, animals were randomized into oral E_2_ supplementation (red bar, 0.3 mg/kg/day for days 8–14, *n* = 18) to achieve significant reductions in T_skin_ values. Finally, two groups of E_2_ rats were randomized to replace the existing E_2_ supplementation with 1 or 3 mg/kg/day ERr 731 supplementation (green bar, days 15–21) for the duration of the study. T_skin_ values remained stable in all animals receiving daily gavage with vehicle during the initial phase. The E_2_ treatment of OVX rats significantly reduced T_skin_ similarly to levels observed in animal studies 1 and 2. ERr 731 replacement therapy at doses of 1 and 3 mg/kg/day lowered T_skin_ similarly to E_2_, although some T_skin_ value rebound was observed at the lower dose tested (Figure 5).

### 2.4. Plasma Levels of Rhubarb Extract Bioactives

**Figure 6:**
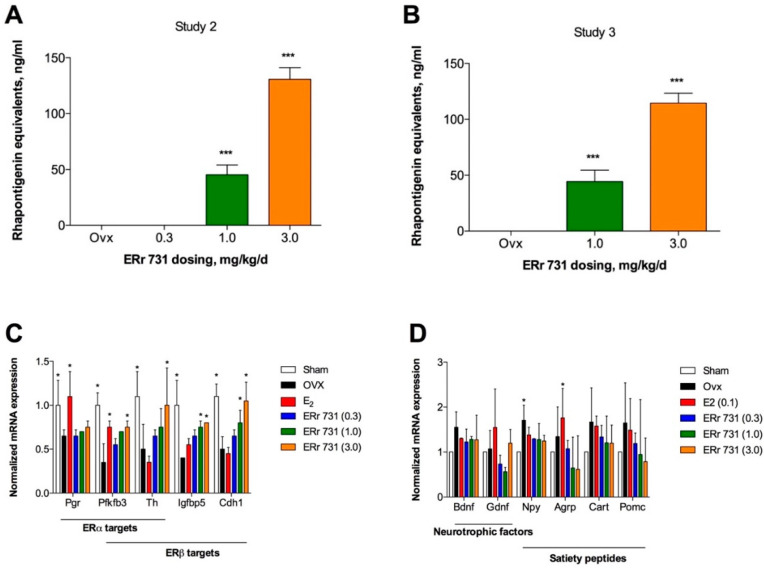
Plasma concentration of ERr 731 metabolite and the pharmacogenomic profiles for ERα/β target genes and bioactive peptides in the hypothalamus of ERr 731-treated rats. (**A**) Rhaponticin metabolite was detected in the plasma of rats treated orally with 0.3–3 mg/kg/day ERr 731 for 14 days in study 2 and (**B**) 7 days in study 3. (**C**) E_2_ treatment significantly upregulated ERα but not ERβ target genes in the hypothalamus of OVX rats, and the opposite effect was observed for ERr 731. (**D**) Neither E_2_ nor ERr 731 modulated the expression of neurotrophic factors; however, an agouti-related peptide *Agrp* was significantly elevated following the OVX surgery and ERr 731 treatment abolished this response. Individual hypothalamic tissue samples (*n* = 6) were analyzed by fluorescent quenching (**A**,**B**) or qPCR (**C**,**D**), and the data were expressed as the mean ± SEM, * *p* < 0.05 and *** *p* < 0.001 vs. OVX control.

Rhaponticin aglycone was detected in the plasma of animals receiving 1 or 3 mg/kg/day ERr 731 botanical extract in the range of 45–130 ng/mL (animal study 2) and 44–114 ng/mL (animal study 3), thus confirming its intestinal absorption and bioavailability (Figure 6A,B).

### 2.5. Hypothalamic Gene Expression Profiles for ERα/β Target Genes and Peptides

ERα/β targets were selected based on a prior global identification of estrogen receptor target genes and signaling networks [19], as well as common neurotrophic factors (*Bdnf*, *Gdnf*) and satiety peptides (*Npy*, *Agrp*, *Cart*, *Pomc*) [20]. Remarkably, E_2_ treatment significantly upregulated the expression of progesterone receptor (*Pgr*), an ERα target gene, and this response was absent in the ERr 731-treated animals. Two putative ERβ target genes (*Igfbp5* and *Cdh1*) showed a significant, dose-dependent response to ERr 731, but not E_2_ treatments (Figure 6C). Neither E_2_ nor ERr 731 modulated the expression of neurotrophic factors in the hypothalamus (Figure 6D). Among the satiety peptides tested, agouti-related peptide *Agrp* was significantly elevated following the OVX surgery and ERr 731 (but not E_2_) treatment abolished this response (Figure 6D).

### 2.6. ERr 731 Shows a Selective ERβ Agonist Activity In Vitro

**Figure 7:**
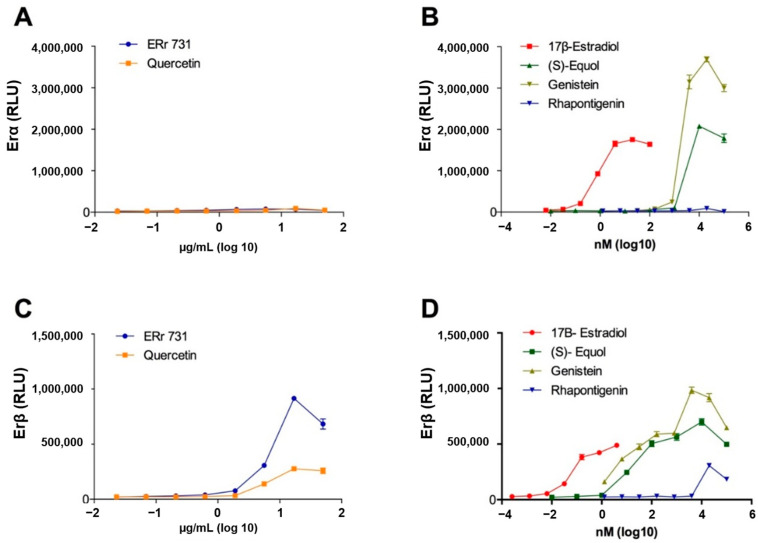
Induction of ER-α (**A**,**B**) activity versus ER-β activity (**C**,**D**) from ERr 731 extract as compared to quercetin, estradiol, equol, genistein and rhapontigenin. ERα or ERβ reporter cells were treated in triplicate wells with various concentrations of test compounds for 24 h, and ER receptor activity was quantified as relative luminescence units (RLU). Agonist concentrations were transformed to log10 µg/mL for botanical extracts or log10 nM for single compounds. Each data point represents mean ± SD.

ERr 731 had little effect on ERα, causing a less than 3-fold increase over the maximum reported activity (Figure 7A), but strongly activated ERβ to a 36-fold increase over the maximum reported activity (Figure 7B). Similar to genistein and (S)-equol, ERr 731 and its major bioactive aglycone, rhapontigenin, acted as full ERβ agonists (Figure 7C,D). Overall, ERr 731 showed a 13.5-fold difference in ERβ/ERα specificity when compared to E_2_ at a maximum receptor activation value. The specificities for the other known estrogenic compounds were much smaller, in the range of 0.31 for E_2_, 0.37 for equol, 0.3 for genistein, and 3.2 for quercetin.

## 3. Discussion

The top eleven major health issues present in peri- and postmenopausal women are vasomotor symptoms, depression, dementia, sleep disorders, migraine, cardiovascular disease, metabolic syndrome, diabetes, chronic respiratory syndrome, cancer and musculoskeletal diseases [2]. Vasomotor symptoms are tightly linked with hormonal loss and the quality of life is severely affected. Since there is a large placebo effect in humans, and the majority of the tested natural compounds showed an inconsistent result in clinical studies for the alleviation of vasomotor symptoms [5], there is a critical need for the development of proper screening methods that predict the efficacy of such therapeutic intervention in humans.

In this study, we used a noninvasive method for measuring T_skin_ with SubCue Mini data loggers in a modified protective casing, attached to the surface of the rat tail [17]. Because T_skin_ increase highly correlates with the occurrence of hot flushes, it has been commonly used as one of the phenotypical (objective) markers for menopause symptoms [21]. In OVX rats, vasodilation (T_skin_ increase) starts at a significantly lower core temperature than in Sham rats [22]. This is a strong indication that OVX rats are more prone to undergo induction of vasodilatory heat dissipation, and therefore provide a good model for studying menopausal hot flashes [23]. This technology is readily accessible to most laboratories and, in our opinion, represents a highly suitable screening platform to evaluate dietary, botanical, or pharmaceutical interventions for their ability to modulate the mechanisms and treatments of menopause.

Our experimental findings provide further evidence for the efficacy of a rhubarb root extract ERr 731 in improving vasomotor menopausal symptoms, and raise the intriguing possibility that it is effective in improving wellbeing during the menopausal transition. E_2_ treatment (0.1 mg/kg/day) of OVX rats resulted in decreased T_skin_, which was evident on day 1 and reached significance by the dark phase of day 2, similar to the previously published studies [18]. In a dose ranging study, ERr 731 botanical extract administered at low (0.3 mg/kg/day), middle (1 mg/kg/day), or high (3 mg/kg/day) doses reduced the T_skin_ values of OVX rats by an average of 1 °C. The human equivalent dose for the middle dose used in this study was estimated at 0.16 mg/kg/day or 10 mg/day of ERr 731 extract for an average adult [24]. This is in line with previous uses of ERr 731 in humans to alleviate the symptoms of menopause. The ERr 731 group receiving 4 mg/day of the botanical extract showed significant improvements in 11 common menopausal complaints during a 12-week randomized, double-blind clinical study [7]. In a 6-month observational study, ERr 731 taken at 4 or 8 mg/day resulted in a significant decrease in the menopause rating scale total score, from 14.5 points at baseline to 6.5 points [13]. This compares favorably to the dosing of soy isoflavones (30–90 mg/day) [25], red clover isoflavones (40–120 mg/day) [26] or black cohosh triterpene glycosides (10–60 mg/day) [27], to achieve similar beneficial results.

The present study was also designed to examine ERr 731 supplementation after hormone replacement therapy (HRT) withdrawal or the discontinuation of E_2_ treatment. ERr731 supplementation at 1 and 3 mg/kg/day after E_2_ withdrawal lowered T_skin_ similarly to E_2_, and this effect was set within 2 days of treatment. Substituting ERr 731 after E_2_ withdrawal therefore helped maintain body temperature similarly to E_2_ alone, suggesting the usefulness of ERr 731 as an alternative approach to existing hormonal therapy in humans. The oral bioavailability of ERr 731 was further confirmed by measuring the plasma levels of its primary metabolite, rhapontigenin.

Because hot flushes correlate with spikes in the peripheral LH and GnRH released from the hypothalamus, they most likely represent a disorder of hypothalamic thermoregulation [19]. In order to substantiate the T_skin_ changes with the hypothalamic expression patterns of genes related to the ERα/β signaling and bioactive peptides, we performed a pharmacogenomics assessment of gene expression profiles in the hypothalamus of OVX rats treated with E_2_ or ERr 731. We observed a prominent ERβ selectivity of ERr 731 in vivo, as the treatment was associated with the activation of ERβ target genes (*Th*, *Igfbp5* and *Cdh1*), and had little effect on the ERα-specific expression profiles. The ERβ specificity was further confirmed in reporter assays, as ERr 731 and rhapontigenin (an active metabolite of rhaponticin) acted as the full agonist of the ERβ receptor. Both the extract and the pure compound were highly selective for ERβ, compared to E_2_ and other estrogenic compounds, such as genistein and equol. These results were consistent with a previous study, where the total ERr 731 extract, as well as its individual compounds, have been demonstrated to act as potent, selective ERβ agonists in human endometrial cells, without affecting ERα-meditated activities [9]. While no changes in the expression levels of the hypothalamic neurotrophic factors were observed in the ERr 731-treated animals, ERr 731 (but not E_2_) treatment had a moderate suppressive effect on the mRNA levels of the agouti-related peptide *Agrp*. This might explain the small but significant reduction in body weight and fat mass associated with the administration of 1 mg/kg/day ERr 731, but the effect was not dose-dependent. AgRP is a crucial part of the melanocortin system involved in the regulation of food intake and energy balance through antagonistic effects on the melanocortin 3 and 4 receptors that stimulate a long-lasting increase in food intake [28]. Other botanical interventions, for example steroidal glycosides from milkweeds, were also shown to modulate AgRP levels in vivo [29].

In summary, this study validated the OVX/Tskin rat model as a suitable screening platform to evaluate the effects of dietary and botanical interventions on the mechanisms and treatments of menopause. Our experimental findings provided further evidence for the efficacy of ERr 731 in alleviating vasomotor menopausal symptoms, and raised the intriguing possibility of its effectiveness in improving wellbeing during the menopausal transition. Substituting ERr 731 after E2 withdrawal helped maintain body temperature similarly to E_2_ alone, suggesting the usefulness of ERr 731 supplementation after the termination of HRT if these findings are confirmed in the future clinical studies.

## 4. Materials and Methods

### 4.1. Chemicals

The ERr 731 botanical extract from the Siberian rhubarb, also known as rhapontic rhubarb (*Rheum rhaponticum* L., family Polygonaceae), was manufactured by Chemisch-Pharmazeutische Fabrik Göppingen Carl Müller Apotheker GmbH & Co. (Göppingen, Germany) (Figure 1), and kindly provided by Metagenics Inc. (Gig Harbor, WA, USA). Rhapontigenin, (S)-equol and genistein were purchased from Cayman Chemicals (Ann Arbor, MI, USA). All other chemical reagents including 17β-estradiol (E_2_) and solvents (analytical grade) were purchased from Sigma (St Louis, MO, USA).

### 4.2. Monitoring of Tail Skin Temperature T_skin_

To record T_skin_, SubCue Mini data loggers (Canadian Analytical Technologies, Calgary, AB, Canada) were inserted into a protective covering (University Research Instrumentation Center, University of Arizona, Tucson, AZ, USA) as described previously [17] with the following modification: the length of the covering was extended by 5 mm to provide additional protection for data loggers against accidental nibbling. The assembled data loggers were attached on the ventral surface of the tail using double-sided tape. The data loggers were calibrated and set to record temperature every 15 min for the duration of the study. Mean T_skin_ values were calculated separately for the dark (active) and light (non-active) phases of the day cycle. All animal experiments were performed according to procedures approved by the NC Research Campus Institutional Animal Care and Use Committee in the David H. Murdock Research Institute, the AAALAC accredited animal care facility, and followed NIH guidelines.

### 4.3. Animal Study 1: Model Validation

Sham-operated and bilaterally ovariectomized female Sprague Dawley rats (10-week-old, 200–250 g, Charles River Laboratories, Wilmington, MA, USA) were housed 2 per cage in a temperature- (21–23 °C) and humidity-controlled environment under an inverted 12 h light cycle (lights on at 1900 h). Rats were fed an AIN-93 standard diet (Research Diets, New Brunswick, NJ, USA) and tap water ad libitum. The experimental groups (*n* = 6) consisted of (1) sham-operated controls gavaged daily with 1 mL of inactive vehicle (10% DMSO in saline), (2) OVX controls gavaged daily with the vehicle, (3) OVX, gavaged daily with 0.1 mg/kg/day E_2_ suspension in vehicle, and (4) OVX, gavaged daily with 1 mg/kg/day ERr 731 suspension in vehicle. T_skin_ temperature recordings were obtained daily for the duration of the study. On day 8, the animals were fasted overnight, sacrificed, and a terminal blood sample was collected via cardiac puncture.

### 4.4. Animal Study 2: Dose Response

The experimental groups (*n* = 6) consisted of (1) OVX controls gavaged daily with vehicle (10% DMSO in saline); (2) OVX, gavaged daily with 0.1 mg/kg/day E_2_ suspension in vehicle; (3–5) OVX, gavaged daily with either 0.3, 1.0, or 3.0 mg/kg/day ERr 731 suspension in vehicle. T_skin_ temperature recordings were obtained during the 12 h dark (active) phase of the light cycle on days 2, 7 and 14 of the study. On day 16, animals were subject to body composition measurements by quantitative magnetic resonance using EchoMRI (Echo Medical Systems, Houston, TX, USA). Unanesthetized rats were placed in plastic restrainer tubes, and triplicate measurements were completed in less than 5 min. Next, animals were fasted overnight, sacrificed on day 17, and a terminal blood sample was collected via cardiac puncture. Terminal body weights, organ weights, and tissues samples were taken at each scheduled sacrifice. Tissues and serum were stored at −80 °C until molecular assays were performed. This study was also repeated once with similar results (not shown).

### 4.5. Animal Study 3: Replacement Therapy

All experimental groups (*n* = 6) were subjected to the treatment period set at 21 days and divided in three 7-day periods: days 1–7 (initial period, all animals received daily gavage with vehicle 10% DMSO in saline); days 8–14 (E_2_ supplementation period, all animals except OVX controls received daily gavage with 0.3 mg/kg/d E_2_ suspension in vehicle); and days 15–21 (replacement therapy period, E_2_ animals were further randomized into three groups and continued to receive either 0.3 mg/kg/d E_2_ or 1–3 mg/kg/day ERr 731 suspension in vehicle as a replacement treatment).

### 4.6. Quantification of Plasma Rhaponticin

Rhaponticin was measured in OVX rat plasma collected from animal studies 2 and 3. In both experiments, the last dosing was performed 1 h prior to animal sacrifice and plasma collection [30]. The presence of rhaponticin in plasma was detected by the fluorescent quenching method in the presence of 10 μM cerium nitrate probe (pH 7.4), as described previously [31].

### 4.7. Gene Expression Studies

Gene expression profiles for ERα/β target genes [32] as well as neurotrophic factors (*Bdnf*, *Gdnf*) and satiety peptides (*Npy*, *Agrp*, *Cart*, *Pomc*) [29] were measured by real-time quantitative PCR (qPCR) in hypothalamus brain tissues collected from animal study 2. The total RNA was isolated using TRIzol reagent (Life Technologies, Carlsbad, CA, USA). RNA was quantified using the SynergyH1/Take 3 spectrophotometer (BioTek, Winooski, VT, USA). The cDNAs were synthesized using 2 µg of RNA for each sample using a high-capacity cDNA Reverse Transcription kit on the ABI GeneAMP 9700 (Life Technologies).

The resulting cDNAs were amplified by qPCR using SYBR green PCR Master Mix (Life Technologies). To avoid interference due to genomic DNA contamination, only intron-overlapping primers were selected using the Primer Express version 2.0 software (Applied Biosystems, Foster City, CA, USA). qPCRs were performed on an ABI 7500 Fast (Life Technologies) using 1 cycle at 50 °C for 2 min and 1 cycle of 95 °C for 10 min, followed by 40 cycles of 15 s at 95 °C and 1 min at 60 °C. The dissociation curve was completed with 1 cycle of 1 min at 95 °C, 30 s at 55 °C, and 30 s at 95 °C. mRNA expression was analyzed using the 2^-ΔΔ**C**T^ method [20] and normalized with respect to the expression of the *gapdh* housekeeping genes using 7500 Fast System SDS Software v1.3.0 (Life Technologies). The amplification of specific transcripts was further confirmed by obtaining melting curve profiles.

### 4.8. Estrogen Receptor Activity

ERα/ERβ receptor assays were performed by Indigo Biosciences (College Park, PA, USA). The ERα/ERβ reporter vectors used in these studies comprised the firefly luciferase gene functionally linked to the appropriate upstream ER response element. Cells were treated in triplicates with various concentrations of test articles, including ERr 731 (0.023–50 µg/mL), rhapontigenin (0.01–100 µM), quercetin (0.023–50 µg/mL), genistein (0.001–100 µM) and (S)-equol (0.00001–100 µM). E_2_ was used as positive control and DMSO (0.1%) was used as a solvent control. Cells were incubated for 24 h and average relative light unit (RLU) values were used to determine ERα and ERβ activity.

### 4.9. Statistics

Data were analyzed by one-way ANOVA followed by Dunnett’s multiple-range tests using Prism 6.0 (GraphPad Software, San Diego, CA, USA). All data were presented as means  ±  SEM. Significant differences were accepted when the *p*-value was <0.05.

## Data Availability

The data presented in this study are available on request from the corresponding author.

## Abbreviations

E_2_	17β-estradiol
ERr 731	Siberian rhubarb *Rheum rhaponticum* L. root extract
ERα/β	Estrogen receptor α/β
HRT	Hormone replacement therapy
OVX	Ovariectomized
T_skin_	Skin temperature
